# Sea Cucumber Polysaccharide from *Stichopus japonicu* and Its Photocatalytic Degradation Product Alleviate Acute Alcoholic Liver Injury in Mice

**DOI:** 10.3390/foods13060963

**Published:** 2024-03-21

**Authors:** Haoran Song, Chen Song, Chunhong Yan, Jingfeng Yang, Shuang Song

**Affiliations:** SKL of Marine Food Processing & Safety Control, National Engineering Research Center of Seafood, Liaoning Key Laboratory of Food Nutrition and Health, School of Food Science and Technology, Dalian Polytechnic University, Dalian 116034, China; 18104099219@163.com (H.S.); sczoe86@163.com (C.S.); chyan@dlpu.edu.cn (C.Y.); yjfgo@163.com (J.Y.)

**Keywords:** polysaccharide, alcohol drink, alcoholic liver disease, sea cucumber, lipid metabolism

## Abstract

To prevent alcoholic liver disease, the addition of bioactive substances to the alcoholic drink Baijiu has been considered a feasible option. In the present study, the hepatoprotective effects of a sea cucumber sulfated polysaccharide (SCSP) isolated from *Stichopus japonicu* were investigated. Moreover, in order to enhance its solubility in an alcohol solution, it was depolymerized using a photocatalytic reaction, and the photocatalytic degradation products (dSCSPs) with an average molecular weight of less than 2 kDa were studied and compared with SCSP. They were characterized by a series of chemical and spectroscopy methods and the oligosaccharide fragments in the dSCSP were further identified by HPLC-MSn analysis. Then, the in vivo experiment showed that the addition of SCSP or dSCSP to Baijiu could alleviate alcoholic liver injury in mice. Further analysis also revealed their protective effect in reducing oxidative stress damage and their regulation of the metabolism of phosphatidylcholine (PC) and phosphatidylethanolamine (PE) in the liver. Of note, dSCSP was more effective at reducing the level of malondialdehyde in the liver. These findings indicate that the addition of sea cucumber polysaccharide or its low-molecular-weight derivative in Baijiu has the potential to alleviate alcoholic liver injury.

## 1. Introduction

*Stichopus japonicus*, an edible sea cucumber species, has been used as a nutraceutical food for thousands of years in Asian countries. The sulfated polysaccharide from sea cucumber (SCSP) is considered one of the most critical bioactive components of sea cucumber, and it is mainly composed of fucosan sulfate and fucolysated chondroitin sulfate [1]. In recent years, it has been reported that SCSP has biological activities, such as anti-inflammatory [2], anticancer [3], hypoglycemic [4,5], anticoagulant [6,7], and antioxidant properties [8]. Among them, the in vitro, antioxidant activity of SCSP has been well documented, and previous studies [9,10,11] have shown that SCSP has a strong ability to clear DPPH, hydroxyl, and superoxide radicals. In addition, SCSP could also regulate host gut microbiota [12,13] and metabolism to achieve physiological functions, such as adjusting host blood lipid levels to reduce weight [14].

Baijiu is a general name for grain-brewed wine in China. It is very popular and is consumed in large amounts. However, like other alcoholic beverages, Baijiu may also induce alcoholic liver disease, which poses a great threat to human health [15]. Alcoholic liver disease (ALD) is one of the common liver diseases caused by frequent alcohol consumption. The liver is the main organ responsible for alcohol metabolism, and, during the process of alcohol metabolism, liver cells are inevitably damaged, leading to inflammation, fatty liver cirrhosis, liver fibrosis, and even liver cancer [16]. More and more evidence indicates that oxidative stress is involved in the pathogenesis of alcoholic liver injury [17], as ethanol treatment has been shown to lead to the production of a large amount of intracellular reactive oxygen species (ROS) and a decrease in the content and activity of antioxidants and various oxidative stress-related enzymes, leading to oxidative stress damage in liver cells. Sea cucumber polysaccharides have been reported to enhance the antioxidant capacity of the host by regulating the JNK and ERK [18] signaling pathways, which indicates its potential application in alleviating ALD.

Functional Baijiu, which is prepared by adding nutraceuticals, is always a popular type of Baijiu product in the market, and it is feasible that it could prevent the negative effect of Baijiu on the liver by the addition of some bioactive components. Considering its antioxidant capacity and other beneficial bioactivities, SCSP could be selected as a promising candidate. However, more experimental evidence is still needed to prove its effects. In addition, because the high molecular weight may limit the solubility of SCSP, it is necessary to prepare its low-molecular-weight derivative, whose effects also need to be studied. 

Photocatalytic degradation is a new type of “green” degradation method that has received great attention in recent years. Compared with traditional degradation methods, the process of photocatalytic degradation is more environmentally friendly and has a higher yield [19]. The degradation performance of photocatalysis is attributed to the oxidative degradation reaction induced by a hydroxyl radical (•OH) and TiO_2_. With high chemical stability and low biological toxicity, this reaction can promote the production of a hydroxyl radical to achieve the catalytic degradation process [19]. It has been reported that photocatalytic degradation of sulfated polysaccharides can reduce their molecular weight without the obvious loss of sulfate groups [19].

Therefore, in the present study, a low-molecular-weight derivative of SCSP was prepared by using the photocatalytic method and was analyzed with infrared spectroscopy (IR), nuclear magnetic resonance (NMR), high-performance liquid chromatography-mass spectrometry (HPLC-MS^n^), and other techniques. Then, the present study evaluated the influence on the liver of the addition of dSCSP and SCSP to a 40% alcohol solution through histopathological examination, physiological and biochemical indexes, and changes in liver lipid metabolism. The findings in the present study could provide references to produce functional Baijiu with little negative influence on the liver.

## 2. Material and Methods

### 2.1. Preparation and Degradation

The sea cucumbers (*Stichopus japonicus*) were collected in Dalian, Liaoning province, China. The TiO_2_ (CAS: 13463-67-7), MnO_2_ (CAS: 1313-13-9), *n*-Butanol (CAS: 71-36-3), acetic acid lichenol (CAS: 6153-39-5), and trifluoroacetic acid (CAS:76-05-1) were purchased from Macklin reagent, Shanghai, China. The 1,9-dimethyl methylene blue (CAS: 931415-92-7) and acetonitrile (CAS: 75-05-8) were purchased from Sigma-Aldrich reagent, Shanghai, China. The 1-phenyl-3-methyl-5-pyrazolone (CAS: 89-25-8) and KBr (CAS: 7758-02-3) were purchased from Aladdin, Shanghai, China. The ammonium acetate (CAS: 631-61-8) was purchased from Solarbio, Beijing, China. The 30% hydrogen peroxide (CAS: 7722-84-1) and concentrated sulfuric acid were purchased from Damao Chemical Reagent, Tianjin, China. The D_2_O (CAS: 7789-20-0) was purchased from Leyan Chemical Reagent China, Shanghai.

The method of extracting polysaccharides from sea cucumber was referred to as the ZHU’s method [14]. Briefly, the sea cucumbers were powdered and hydrolyzed with papain, and the sulfated polysaccharide was precipitated with cetyl pyridine chloride to obtain sea cucumber polysaccharide (SCSP).

The degradation of SCSP was carried out based on our previous report [19] and was improved. Briefly, 2 g SCSP, 2 g titanium dioxide (TiO_2_), and 10 mL 30% hydrogen peroxide (H_2_O_2_) were added to 200 mL of distilled water and mixed well. After a 12 h photocatalysis reaction under a Photocatalytic Xenon Light Source System (CEL-HXF300-T3), about 1 g manganese dioxide (MnO_2_) was added and the solution, and it was left to stand overnight to remove residual H_2_O_2_. Then, the supernatant was collected after centrifugation (8000 rpm, 15 min), and anhydrous ethanol was added until the ethanol concentration reached 90%. After overnight standing and centrifugation (8000 rpm, 15 min), the precipitate was collected and was named dSCSP.

### 2.2. High-Performance Gel Permeation Chromatography (HPGPC) Analysis

The molecular weight distribution of the polysaccharide degradation products was measured using HPGPC by a Waters 2414 HPLC-equipped differential refraction detector. TSK-gel G5000PWxl (7.5 mm × 30.0 cm) and TSK-gel G2500PWxl (7.5 mm × 30.0 cm) were applied, and dextran of different molecular weights (670 kDa, 410 kDa, 50 kDa, 25 kDa, 5 kDa) was used as the standard substance. The temperature of the column box was 30 °C, the sample size was 10 μL, the mobile phase was 100 mM ammonium acetate, and the flow rate was 0.4 mL/min.

### 2.3. Thin Layer Chromatography (TLC) Analysis

Thin layer chromatography was a previously reported method [20]. In brief, 5 mg/mL SCSP, dSCSP, and standard products (glucose, lactose, and β-cyclodextrin) were loaded onto a silica gel thin-layer chromatographic plate. *n*-Butanol, acetic acid, and water (2:1:1, *v*/*v*/*v*) were used as the mobile phase. After separation, the thin layer chromatographic plates were air-dried, sprayed with a lichenol/concentrated sulfuric acid/methanol (10:1:5, *v*/*v*/*v*) solution, and then heated at 105 °C for 3 min until color development.

### 2.4. Chemical Composition Analysis

The contents of uronic acid, sulfated polysaccharides, and sulfate groups of SCSP and its degradation products were determined. The content of uronic acid was measured using the *m*-hydroxybiphenyl method. Determination of sulfated polysaccharide content was conducted using 1,9-dimethyl methylene blue. In addition, the gelatin turbidimetric method was used to determine the content of sulfate groups.

### 2.5. Monosaccharide Composition Analysis of SCSP and Its Degradation Products

The monosaccharide composition was analyzed using Qiong’s method [21], with some modifications. Briefly, SCSP and its degradation products were hydrolyzed with 2.0 M trifluoroacetic acid (TFA) at 121 °C for 3 h, then dried by nitrogen and labeled with 1-phenyl-3-methyl-5-pyrazolone (PMP). Then, the PMP derivatives were analyzed using HPLC coupled with a photodiode array (PDA) detector which was controlled by XCalibur software (Thermo Fisher Scientific, Shanghai, China). A Diamosil C18 column (250 mm × 4.6 mm, 5 μm, Dikma Technologies Inc., Beijing, China) was applied with a column temperature of 35 °C and the mobile phase was 20 mM ammonium acetate (A) and acetonitrile (B) (A:B = 78.5:22.5, *v*/*v*) with a flow rate of 1.0 mL/min.

### 2.6. HPLC-PDA-MS^n^ Analysis

After the derivatization with PMP, as mentioned in Section 2.5, the dSCSP was analyzed by an LXQ linear ion trap mass spectrometer equipped with an electrospray ion source (ESI) and a PDA detector, controlled by XCalibur software (Thermo Fisher Scientific, Shanghai, China). The ESI-MS settings were set as we previously reported [22]. A TSKgel-Amide-80 (4.6 mm × 150 mm, 3 μm) column was used, the column temperature was 35 °C, and the mobile phase consisted of 20 mM ammonium acetate-acetonitrile (78:22, *v*/*v*, pH = 6.0) with a flow rate of 0.2 mL/min. The data in positive mode were collected.

### 2.7. Fourier Transform-Infrared (FT-IR) Spectroscopy

SCSP and dSCSP (each 2 mg) were individually mixed with 100 mg dry KBr and pressed into 1 mm pellets. Then, the pellets were individually analyzed by a Spectrum One-B FT-IR Spectrometer (Perkin Elmer, Waltham, MA, USA) in the frequency range of 4000 cm^−1^ to 400 cm^−1^.

### 2.8. NMR Spectroscopic Analysis

SCSP and dSCSP (each 30 mg) were individually dissolved in 500 μL D_2_O (99.9%) and freeze-dried three times. Then, their ^1^H NMR spectra were recorded on a Bruker Ascend 400 spectrometer (Bruker, Bremen, Germany).

### 2.9. Animal Experiments

All experiments were conducted according to the Regulations of Experimental Animal Administration issued by the State Committee of Science and Technology of the People’s Republic of China, and all mouse procedures were reviewed and approved by the Animal Research Ethic Committee of Dalian Polytechnic University (ID DLPU2022072). Female Balb/*c* mice (18–22 g) were purchased from Liaoning Changsheng Biotechnology Co., Ltd. (Changchun, China). All mice were housed in a clean room with no specific pathogens. The clean room was controlled under conditions of 21–25 °C, 50–55% relative humidity, and the dark/light cycle was 12 h. The mice had free access to disinfected feed pellets and distilled water.

After acclimatization to standard laboratory conditions for one week, all the mice were randomly divided into six groups with 10 mice in each group, as shown in Table 1. The control group and model group were gavaged with 0.3 mL distilled water and 0.3 mL mimic Baijiu (40% ethanol), respectively, for 5 days. At the same time, the other groups received different concentrations of SCSP or dSCSP in mimic Baijiu (the dosage is shown in Table 1; H-SCSP 360 mg/kg/bw, L-SCSP 60 mg/kg/bw, H-dSCSP 360 mg/kg/bw, L-dSCSP 60 mg/kg/bw). The body weights of the experimental mice were recorded daily. After 12 h of fasting following the last gavage, blood samples were obtained rapidly from the eyeballs, and all mice were sacrificed immediately. The blood samples were centrifuged (3500 rpm, 15 min) for serum. After being dissected and weighed, part of the liver sample was immediately placed in 4% paraformaldehyde, and the remainder was immediately frozen in liquid nitrogen and stored at −80 °C for further biochemical experiments.

### 2.10. Hepatic Histological Analysis

The liver samples were fixed in 4% paraformaldehyde for 24 h, then dehydrated in ethanol, embedded in paraffin, and cut into 5 μm thick sections. The sections were stained with hematoxylin and eosin (H&E) successively. Finally, images of histopathological alterations were obtained by an inverted microscope (Zeiss, Oberkochen, Germany).

### 2.11. Biochemical Index Detection

Liver samples were mixed with a ratio of liver weight (mg) to 0.9% saline (mL) of 1:9 and homogenized in an ice–water bath. The prepared 10% homogenates were centrifuged at 2500 rpm and 4 °C for 10 min, and the supernatants were tested for the levels of malonaldehyde (MDA) according to the instructions of the relevant commercial kits. Then, the content of total cholesterol (TC), triglyceride (TG), alanine transaminase (ALT), and aspartate aminotransferase (AST) in the mouse serum samples was determined using kits (Jiancheng Bioengineering Institute, Nanjing, China).

### 2.12. Lipidomics Analysis

The liver samples (200 mg) were pretreated with a liquid-liquid methyl tert-butyl ether (MTBE) extraction [23] with slight modification. Briefly, the lipids of the liver samples were extracted using MTBE, dried, and finally reconstituted in a 200 μL mixture of isopropanol/acetonitrile/water (65:30:5, *v*/*v*/*v*), and then subjected to UPLC-MS/MS analysis using a linear ion trap mass spectrometer (ABscix5500, Washington, DC, USA). Detailed conditions for the chromatograph and mass spectrometer were described previously [24,25]. The metabolite results were analyzed by partial least squares (PLS-DA), orthogonal partial least squares (OPLS-DA), and different-weighted contribution value (VIP) using SIMCA (version 14.1) software.

### 2.13. Statistical Analysis

The statistical analysis was performed using SPSS version 21.0 and the experimental data were analyzed using one-way analysis of variance (ANOVA) unless specified otherwise. *p* < 0.05 indicated a statistically significant difference and *p* < 0.01 indicated a highly statistically significant difference. Figures were plotted using GraphPad Prism 8.0 and OriginPro 2021.

## 3. Results

**Figure 1:**
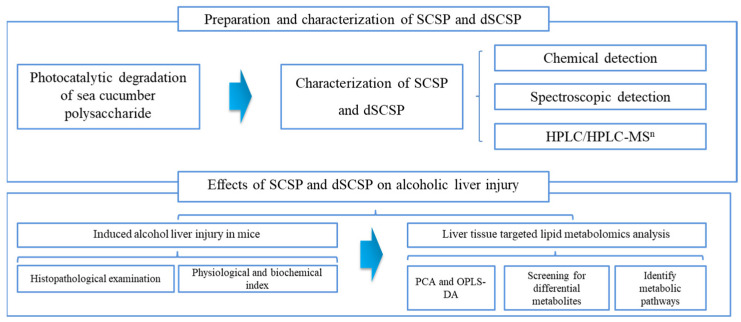
The technical roadmap for this article.

We aimed to study the effect of sea cucumber polysaccharide on alcoholic liver injury and improve its solubility in alcohol. To do this, we obtained low molecular weight sea cucumber polysaccharide by photocatalytic degradation and analyzed its molecular weight, functional group content, monosaccharide composition, structural differences, and oligosaccharide fragments. The protective effects of SCSP and dSCSP on the liver were evaluated by detecting the pathological changes, physiological changes, and lipid metabolism changes in mouse liver. The technical roadmap for this article is shown in Figure 1.

### 3.1. Characterization of SCSP and Its Degradation Products

**Figure 2:**
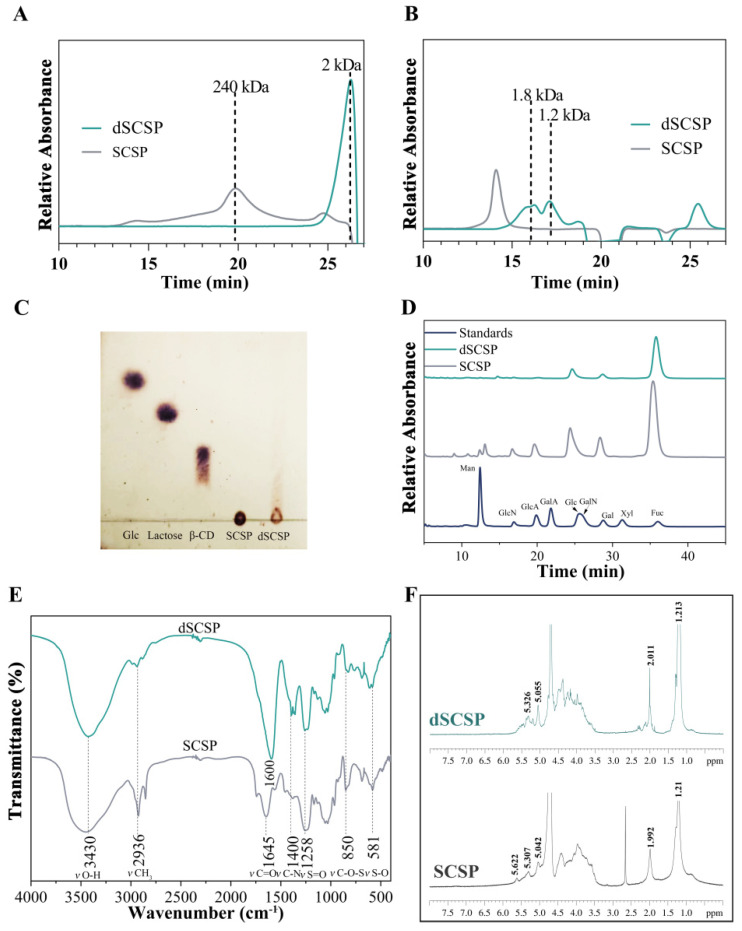
Relative molecular weight of sea cucumber polysaccharide and its photocatalytic degradation products. (**A**) TSK-gel G5000PWxl chromatographic column was used for analysis; (**B**) TSK-gel G2500PWxl chromatographic column was used for analysis; (**C**) thin layer chromatography; (**D**) monosaccharide composition; (**E**) the FT-IR spectrum of SCSP and its photocatalytic degradation products; (**F**) ^1^H NMR spectra of SCSP and dSCSP.

SCSP was extracted from *Stichopus japonicus*, and dSCSP was prepared by photocatalytic degradation followed by ethanol precipitation. As shown by the results of the HPGPC (Figure 2A) on a TSK-gel G5000PWxl column, SCSP only exhibits a peak with a molecular weight of 240 kDa, which indicates it is mainly composed of fucosylated chondroitin sulfate [26]. The average molecular weight of dSCSP was much smaller, and the HPGPC on a TSK-gel G2500PWxl column (Figure 2B) indicates two peaks, suggesting two fractions of 1.8 kDa and 1.2 kDa. In addition, the TLC analysis (Figure 2C) also indicates that dSCSP contains oligosaccharide components.

As shown in Figure 2D, SCSP and dSCSP possessed similar monosaccharide compositions, with Fuc, GalN, and Gal as their major monosaccharide components. Uronic acid, whose content could not be detected accurately by monosaccharide composition analysis due to its instability, was quantified by the m-hydroxybiphenyl method (Table 2), and SCSP and dSCSP showed no significant difference. Moreover, SCSP and dSCSP also possessed similar sulfation degrees.

An FT-IR analysis was also conducted to compare the chemical composition of SCSP and dSCSP (Figure 2E). In the spectrum of SCSP, the absorptions around 1645 cm^−1^ (*ν*_C=O_) and 1400 cm^−1^ (*ν*_C-N_) were derived from GlcA and GalN, respectively, in the chondroitin sulfate structure [14]. Furthermore, the signals at 1258 cm^−1^ (*ν*_S=O_), 850 cm^−1^ (*ν*_C-O-S_), and 581 cm^−1^ (*ν*_S-O_) were derived from sulfate groups [14]. The FT-IR spectrum of dSCSP was similar to that of SCSP. However, it is worth noting that the *ν*_C=O_ absorption peak of dSCSP was shifted to 1600 cm^−1^ and the absorption intensity was slightly increased compared with SCSP, which may be due to the formation of carbonyl group and hydrogen bonds in the process of photocatalytic degradation.

The ^1^H NMR spectra of SCSP and dSCSP are shown in Figure 2F. The signals of SCSP at 1.21 and 1.92 ppm were attributed to the methyl protons of Fuc (CH_3_) and GalNAc (CH_3_CO), respectively, and the signals between 4.50 and 3.40 ppm were attributed to the oxymethylene and methylene protons of the glycoside ring. The signals between 5.65 and 5.00 ppm were attributed to anomeric protons of various sulfated fucose residues. Then, the ^1^H NMR signals of dSCSP indicate that it has similar structural blocks to SCSP with subtle differences.

### 3.2. Identification of Oligosaccharides in dSCSP

**Figure 3:**
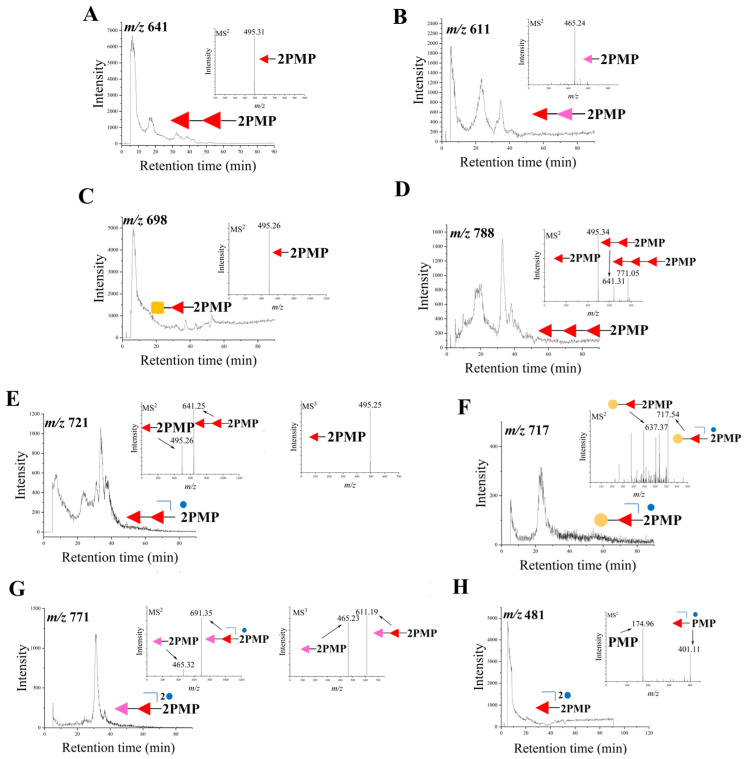
HPLC-MS^n^ analysis results of representative fragments from photocatalytic degradation. The yellow circle (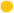) represents dGal, the yellow circle with green border (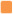) stands for GalNAc, the red triangle (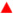) stands for Fuc, the pink triangle (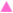) stands for dFuc, and the blue small circle (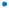) stands for sulfate groups. The product ions at *m*/*z* 641 (**A**), *m*/*z* 611 (**B**), *m*/*z* 698 (**C**), *m*/*z* 788 (**D**), *m*/*z* 721 (**E**), *m*/*z* 717 (**F**), *m*/*z* 771 (**G**), *m*/*z* 481 (**H**).

The oligosaccharides in dSCSP were analyzed by HPLC-MS^n^ after PMP derivatization, and they were elucidated according to the mass spectra and original SCSP composition [14]. As shown in Figure 3A, the pseudo-molecular ion at *m*/*z* 641 [M + H]^+^ gave product ions at *m*/*z* 495 [Fuc + H]^+^ which further yielded a characteristic ion of PMP at *m*/*z* 175, so it was identified as a disaccharide of Fuc→Fuc. Then, its derivative with a 30 Da mass loss has also been observed (Figure 3B), and the pseudo-molecular ion at *m*/*z* 611 [M + H]^+^ gave product ions at *m*/*z* 465 [dFuc + H]^+^, which was proposed as a Fuc linked to 2,3,4-trihydroxypentanal (dFuc) [19]. Fuc→GalNAc or Fuc→GlcNAc was identified (Figure 3C) by the pseudo-molecular ion at *m*/*z* 689 and the product ion at *m*/*z* 495. Furthermore, a trisaccharide fragment Fuc→Fuc→Fuc was also found (Figure 3D). In Figure 3E–G, three sulfated disaccharides, Fuc→Fuc-SO_3_, Gal→Fuc-SO_3_, and Fuc→dFuc-2SO_3_ were characterized by a typical loss of 80 Da. In addition, one sulfated monosaccharide, Fuc-2SO_3_, was also detected (Figure 3H). Thus, a total of eight mono- and oligo-saccharides were identified in dSCSP, including one trisaccharide, three sulfated disaccharides, three disaccharides without sulfate groups, and one sulfated monosaccharide.

### 3.3. Addition of SCSP and dSCSP in Baijiu Reduces Alcoholic Liver Injury

**Figure 4:**
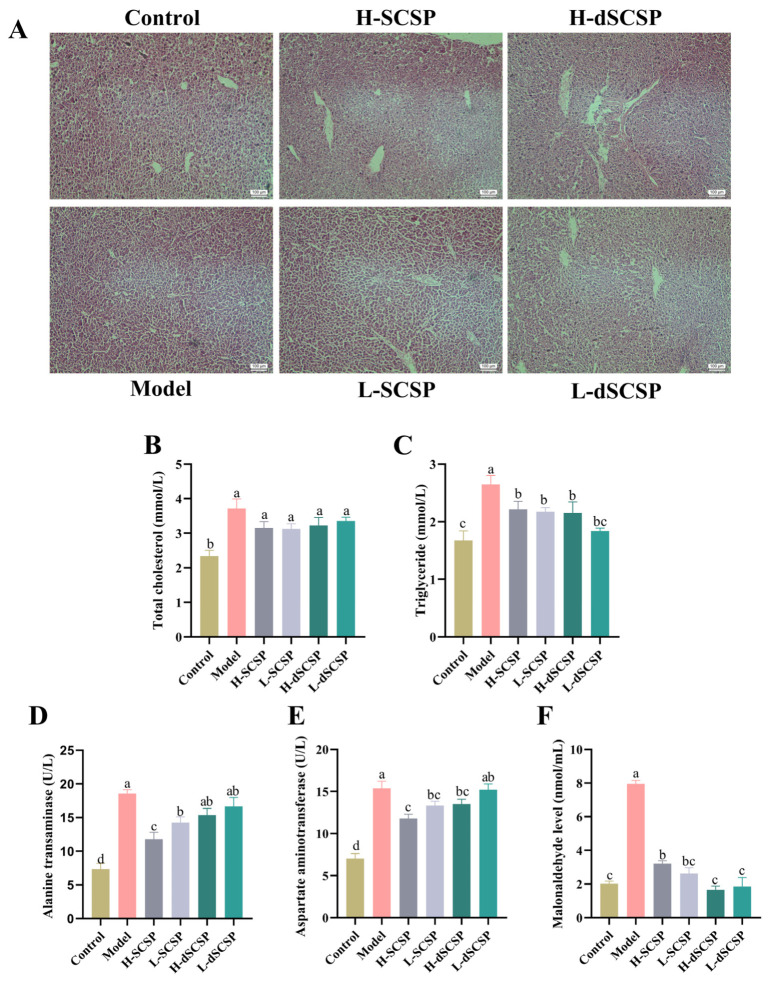
The influence of SCSP and dSCSP on histopathological changes of liver tissue (**A**). The levels of TC (**B**), TG (**C**), ALT (**D**), and AST (**E**) in serum, and the levels of MDA in the liver (**F**), were evaluated. The results are expressed as means ± SEM (n = 6–8). Different letters mean statistically significant differences at the level of *p* < 0.05.

The effects of the Baijiu samples containing SCSP or dSCSP on liver function in mice after 5 days of gavage were investigated. To evaluate the alcoholic liver injury after treatment, histopathological changes in the liver tissues of mice were observed after H&E staining. The analysis method of H&E staining of the liver was referred to in previous reports [27,28]. As shown in Figure 4A, in the control group the hepatocyte cords were arranged in order, the central nucleus was round, and the cord-like structure around the blood vessels could be seen. It is worth noting that the hepatocytes in the model group did not show obvious inflammatory cell infiltration, and the cord-like structure was faintly visible. However, there were no significant differences in the histopathological images among these groups.

The levels of TC and TG in serum are common indicators for liver fat accumulation, while the levels of ALT and AST in serum can reflect the occurrence of inflammation in the liver. As shown in Figure 4B,C, the levels of TC and TG in the serum of the model group mice increased by 59% and 58% compared to the control group, indicating the accumulation of fat in the liver following Baijiu treatment. However, the TC (*p* > 0.05) and TG (*p* > 0.05) levels were reduced in the four groups treated with Baijiu containing different concentrations of SCSP and dSCSP at a certain degree as compared to the model group. Additionally, the levels of ALT and AST (Figure 4D,E) in the model group increased by 153% and 119% compared to the control group, indicating that ethanol induced inflammation in the liver. However, compared with the model group, the levels of ALT and AST in the groups treated with the Baijiu samples containing SCSP all decreased. Among them, the effect of a high dosage of SCSP was the most obvious, with decreases of 37% for ALT and 23% for ALT. As compared with the control group, Baijiu caused alcoholic liver injury and increased the levels of TC, TG, and transaminase in the model group, while those containing different concentrations of SCSP or dSCSP reduced this injury and protected the liver to a certain degree.

During the process of alcohol-induced oxidative damage in liver cells, a large amount of reactive oxygen species (ROS) are produced, causing lipid peroxidation of the liver cell membrane, leading to an increase in the content of the final product of peroxidation, MDA. As shown in Figure 4F, the MDA content in the model group increased by 293% compared to that in the control group, indicating that alcohol increased the amount of oxidative free radicals in the liver and caused further oxidative damage to liver cells. However, the MDA content in the liver of the other four groups treated with Baijiu samples containing SCSP or dSCSP did not increase as remarkably as that of the model group, and the L-SCSP, H-dSCSP, and L-dSCSP groups showed no significant differences with the control group.

### 3.4. Regulation of SCSP/dSCSP on Hepatic Lipid Metabolism

**Figure 5:**
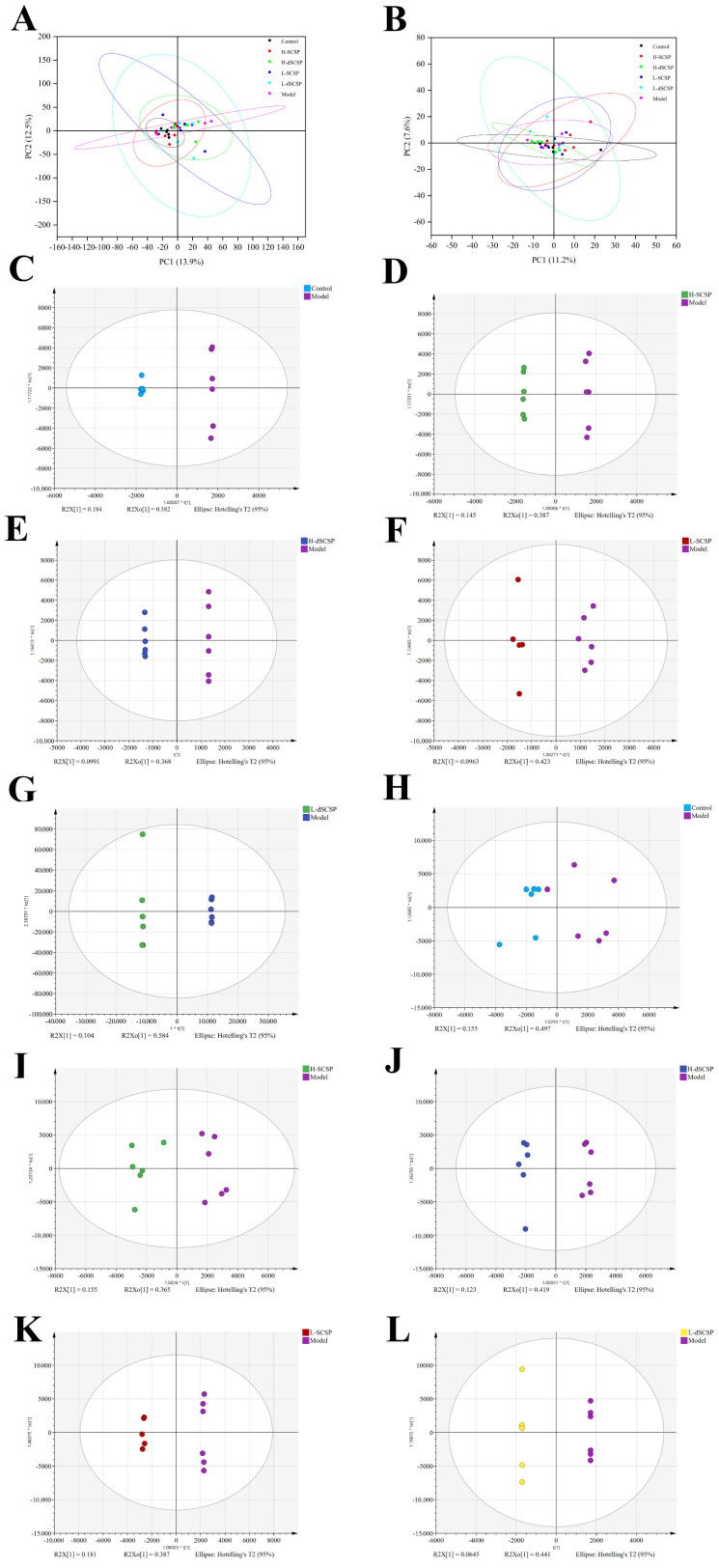
PCA score plots in positive (**A**) and negative (**B**), OPLS-DA score plots between control group and model group in positive (**C**), H-SCSP group and model group in positive (**D**), H-dSCSP group and model group in positive (**E**), L-SCSP group and model group in positive (**F**), L-dSCSP group and model group in positive (**G**), control group and model group in negative (**H**), H-SCSP group and model group in negative (**I**), H-dSCSP group and model group in negative (**J**), L-SCSP group and model group in negative (**K**), and L-dSCSP group and model group in negative (**L**).

To study the preventive effect of the addition of SCSP or dSCSP in Baijiu on the liver function of mice, a lipid metabolomics analysis was conducted with UPLC-MS/MS. The PCA analysis results of the metabolites detected in positive and negative modes are shown in Figure 5A,B, respectively. The cluster of the control group was significantly separated from the model group, which indicated that drinking Baijiu caused changes in lipid content. Of note, the location of the H-SCSP group was closer to the control group, indicating that high-dose SCSP could more effectively ameliorate the abnormal lipid metabolism of the liver caused by drinking Baijiu. The supervised OPLS-DA score plot showed metabolic differences between the control group vs. model group, H-SCSP group vs. model group, H-dSCSP group vs. model group, L-SCSP group vs. model group, and L-dSCSP group vs. model group. Each group was clustered, exhibiting good repeatability (Figure 5C–L). In addition, the results of 200 iterations confirmed that these OPLS-DA models were fitted and had good predictability (Figure S1).

**Figure 6:**
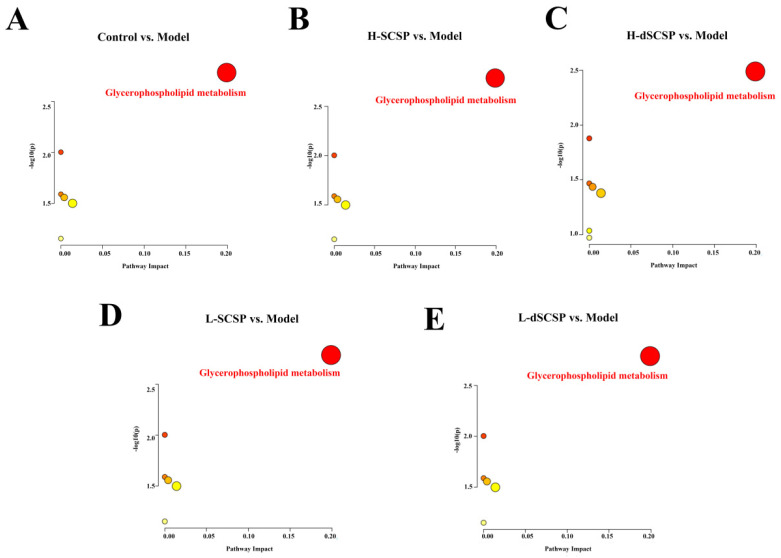
Pathway analysis of the differential lipids, control group vs. model group in positive (**A**), H-SCSP group vs. model group in positive (**B**), H-dSCSP group vs. model group in positive (**C**), L-SCSP group vs. model group in positive (**D**), L-dSCSP group vs. model group in positive (**E**). The color and size of each circle represented the *p*-value and the pathway impact factor (the darker the color, the greater the *p*-value; the larger the bubble, the greater the impact factor), respectively. The pathways written in red were greatly influenced. MetaboAnalyst 3.5 was applied to achieve this function.

**Figure 7:**
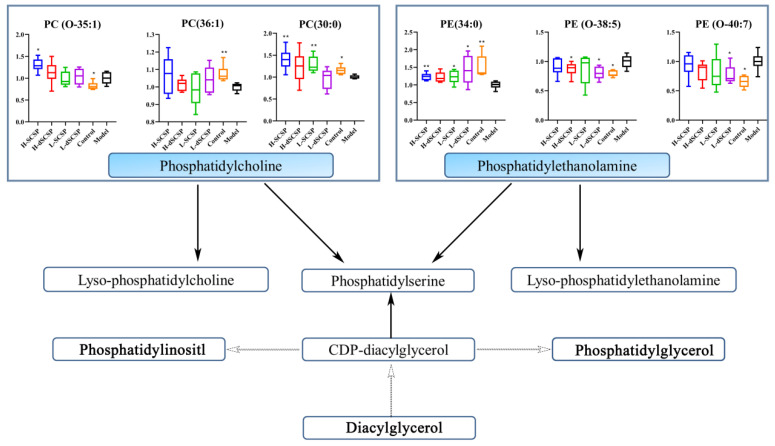
Lipid metabolic pathways regulated by SCSP and dSCSP in liver. * *p* < 0.05 and ** *p*< 0.01 compared with model group.

Furthermore, differential metabolism (VIP > 1) was screened in the OPLS-DA analysis and compared between the control group and model group, and *p* < 0.05 was employed to further screen significantly different metabolites. Then, more than 300 differential lipids (they contain isomers and were not considered the same metabolite) were finally identified, and their heat map is demonstrated in Figure S2. The pathway enrichment analysis based on the regulation of lipid differential metabolites (VIP > 1) revealed glycerophospholipid metabolism as the main pathway involved (Figure 6). Further analysis demonstrated that both SCSP and dSCSP could significantly reverse the abnormal metabolism of phosphatidylcholine (PC) and phosphatidylethanolamine (PE) in the liver that was induced by the Baijiu treatment. As shown in Figure 7, alcohol significantly changed the levels of PE (34:0), PE (O-38:5), PE (O-40:7), and PC (30:0) in the liver. Specifically, the liver PE (34:0) level in the control group was significantly higher than that in the model group, and it could be reduced by SCSP treatment. Meanwhile, dSCSP could significantly improve the abnormal levels of PE (O-38:5) and PE (O-40:7) that are induced by alcohol consumption. Moreover, a high dose of SCSP or dSCSP can effectively reverse the down-regulation of PC (30:0) content in the liver that is caused by alcohol consumption.

## 4. Discussion

Long-term drinking is the direct cause of alcohol-induced liver injury, which is caused by oxidative stress injury of the liver and lipid metabolism dysfunction caused by alcohol. How to protect the health of the liver in the process of drinking alcohol is our current research focus. As a natural bioactive substance with great potential, sea cucumber polysaccharide has been evaluated for its application in preventing liver injury when employed as an additive to Baijiu or other alcoholic drinks.

Reducing the molecular weight of polysaccharides could improve their solubility in Baijiu, so, SCSP was depolymerized by photocatalysis reaction to obtain dSCSP with a molecular weight of less than 2 kDa. The characterization results suggest that the structural blocks of dSCSP are nearly identical to those of SCSP. This is consistent with previous reports that photocatalytic degradation could reduce molecular weight without the obvious loss of functional groups [19].

As we know, the liver is the main organ involved in the metabolism of alcohol in the human body. With the action of ethanol dehydrogenase, ethanol is metabolized into the highly toxic metabolite, acetaldehyde, which is transformed into acetic acid by acetaldehyde dehydrogenase and is ultimately degraded into water and carbon dioxide. During this process, ethanol and acetaldehyde both cause damage to the body through different mechanisms [29,30,31]. Firstly, alcohol can directly promote ROS-induced oxidative stress during the metabolic process, leading to the consumption of a large number of active antioxidant substances in the body, peroxidation reactions, and fat accumulation in the liver [32,33]. Subsequently, liver cells are subjected to a series of oxidative stress reactions and attacks by peroxides and inflammatory factors, resulting in liver cell damage, necrosis, inflammation, and even fibrosis [34]. When the liver’s lipid metabolism ability is impaired, a large amount of lipid accumulation occurs in the liver. MDA is the degradation product of polyunsaturated fatty acid peroxide and has strong toxicity after cross-linking with lipoprotein, which will damage the cell membrane structure [35].

In the present study, both SCSP and dSCSP showed the potential to alleviate acute alcoholic liver injury. The significant increase in ALT and AST content in the serum of the model group mice indicated the occurrence of liver injury, and the addition of SCSP or dSCSP in the Baijiu solution could alleviate the occurrence of liver injury in a dose-dependent manner. Moreover, SCSP was more effective in alleviating the levels of two transaminases released into the blood. The contents of TC and TG could reflect the degree of fat accumulation or dysfunction of lipid metabolism in the liver of mice, and the results showed that both SCSP and dSCSP had the effect of reducing liver lipid levels in mice. Jiang et al. [36] found that *Poria cocos* polysaccharide had a positive therapeutic effect on ALD by reducing the release of inflammatory factors and inhibiting oxidative stress and cell apoptosis. Wang et al. [37] found that a polysaccharide from *Coriolus versicolor* mycelia played a protective role in ALD by reducing oxidative stress by increasing the expression of SOD and CAT. Their study showed that polysaccharides from Coriolus versicolor mycelia in a concentration of 80~160 mg/kg/day could significantly reduce the levels of serum ALT and AST induced by alcohol, and the activity of the SOD and CAT enzymes in the liver were also significantly improved.

The concentration of one of the typical oxidative stress-related indicators, MDA, was significantly increased in the liver of the model group mice. Obviously, alcohol induced oxidative stress damage in the livers of the mice, while both SCSP and dSCSP significantly inhibited the occurrence of such damage in a dose-dependent manner. It is worth noting that dSCSP could more effectively reduce the MDA level in the liver compared to SCSP. Thus, the results indicated that molecular weight affected the oxidative stress activity of sea cucumber polysaccharides. Peng et al. [38] obtained the degradation products of *Porphyra yezoensis* polysaccharides with different molecular weights through H_2_O_2_ degradation and observed the same trend when studying its oxidative stress activation on human renal epithelial cells. Fang et al. [39] found that the enzymatic degradation product of the *Gracilariopsis lemaneiformis* polysaccharide had a better effect in alleviating oxidative damage in HFL1 cells compared to the original polysaccharide. Song et al. [40] found that 600 mg/kg/bw of polysaccharides from *Pleurotus geesteranus* (IMPP) could significantly reduce the heightened level of MDA in the liver induced by alcohol consumption, indicating that IMPP could ameliorate alcohol-induced liver injury by alleviating oxidative stress damage in the liver, which is consistent with our experimental results.

The liver is an important organ of lipid metabolism, and the disorder of lipid metabolism suggests the decline of liver function. The metabolic pathway of glycerophosphate plays an important role in lipid regulation, metabolic regulation, signal transduction, and other physiological functions of the liver. PC and PE are two important lipid metabolites of glycerolphosphate, which can be further hydrolyzed to free fatty acids and lysophospholipids in vivo. Previous reports suggested that changes in PC and PE levels could reflect the disorder of energy metabolism or lipid metabolism in the liver [41]. In the present study, SCSP and dSCSP regulated the abnormal changes in liver PC and PE content caused by alcohol, through the glycerophosphate metabolism pathway, to protect the liver in mice. Similar effects of other polysaccharides and glycosides have also been reported. Bian et al. [42] found that *Mori Fructus* polysaccharide could protect against alcohol-induced damage by regulating the liver PC content and liver glycerophosphate metabolism in rats. Lei et al. [43] reported that a verbascoside played a role in reducing lipid deposition by regulating the metabolism of glycerophospholipids in the liver. Wang et al. [44] showed that Dendrobium huoshanense polysaccharide (DHP) could prevent alcohol-induced liver injury by improving ethanol-induced lipid metabolism disorders in the liver and that DPH mainly regulated the level of phosphatidylcholines (PC) in the mouse liver, which is in line with our findings.

## 5. Conclusions

SCSP extracted from sea cucumbers was degraded by a photocatalytic reaction to obtain dSCSP with an average molecular weight of less than 2 kDa. The oligosaccharides in dSCSP were identified as sulfated disaccharides and trisaccharides composed mainly of fucose and a small amount of galactose and n-acetyl galactose amine. Both SCSP and dSCSP can alleviate oxidative stress damage to the liver caused by alcohol consumption in a dose-dependent manner, thereby alleviating acute alcoholic liver injury. It is worth noting that low molecular weight dSCSP has a better ability to alleviate oxidative damage. Meanwhile, lipid metabolism analysis showed that both SCSP and dSCSP played a protective role in the liver by regulating glycerophospholipid metabolism. In the present study, the findings from the comparison of the effects of SCSP and dSCSP enhance the understanding of the relationship between molecular weight and the liver protective effects of sea cucumber polysaccharide, and the results also suggest that the addition of SCSP or its low-molecular-weight derivative to Baijiu are a potential way to prevent alcoholic liver disease.

The effects of a sea cucumber polysaccharide with a different molecular weight on physiological and biochemical indexes and liver lipid metabolism in mice with acute alcoholic liver injury were studied in the present study. However, the signal pathways regulated by the sea cucumber polysaccharide are still unclear. The antioxidant and anti-inflammatory activities of the sea cucumber polysaccharide on hepatocytes are not well studied. Therefore, more efforts are still needed to reveal the mechanism underlying the protective effect of the sea cucumber polysaccharide on alcoholic liver injury.

## Figures and Tables

**Table 1:** Groups of experimental animals.

Group	Experimental Treatments			
	Distilled Water (mL)	SCSP in 40% Mimic Baijiu (mg/kg Body Weight)	dSCSP in 40% Mimic Baijiu (mg/kg Body Weight)	40% Mimic Baijiu (mL)
Control	0.3	—	—	—
Model	—	—	—	0.3
H-SCSP	—	360	—	0.3
L-SCSP	—	60	—	0.3
H-dSCSP	—	—	360	0.3
L-dSCSP	—	—	60	0.3

**Table 2:** Chemical composition analysis.

	Uronic Acid Content (%)	Sulfate Group Content (%)	Monosaccharide Composition (GlcN:GalN:Gal:Fuc)
SCSP	15.09 ± 1.28	13.10 ± 0.69	2.79:1.00:2.43:17.82
dSCSP	13.08 ± 0.40	11.57 ± 1.09	1.00:1.48:1.93:32.35

## Data Availability

The original contributions presented in the study are included in the article/Supplementary Materials, further inquiries can be directed to the corresponding author.

## Supplementary Materials

The following supporting information can be downloaded at: https://www.mdpi.com/article/10.3390/foods13060963/s1, Figure S1: Permutation test of each group and control group under positive and negative ions ((A–E): positive ion mode, (G–K): negative ion mode), control group vs. model group (A,G), H-SCSP group vs. model group (B,H), H-dSCSP group vs. model group (C,I), L-SCSP group vs. model group (D,J), L-dSCSP group vs. model group (E,K); Figure S2: Heat map of lipid metabolites difference between each group and model group (VIP > 1).
