# Supplementary material for: Sea Cucumber Polysaccharide from Stichopus japonicu and Its Photocatalytic Degradation Product Alleviate Acute Alcoholic Liver Injury in Mice
Source: Foods. 2024 Mar 21;13(6):963. doi: 10.3390/foods13060963 (PMC10969942; doi:10.3390/foods13060963)
Supplement: Supplementary file 1 [file foods-13-00963-s001.zip › foods-2899882-supplementary.pdf]

Supplementary materials

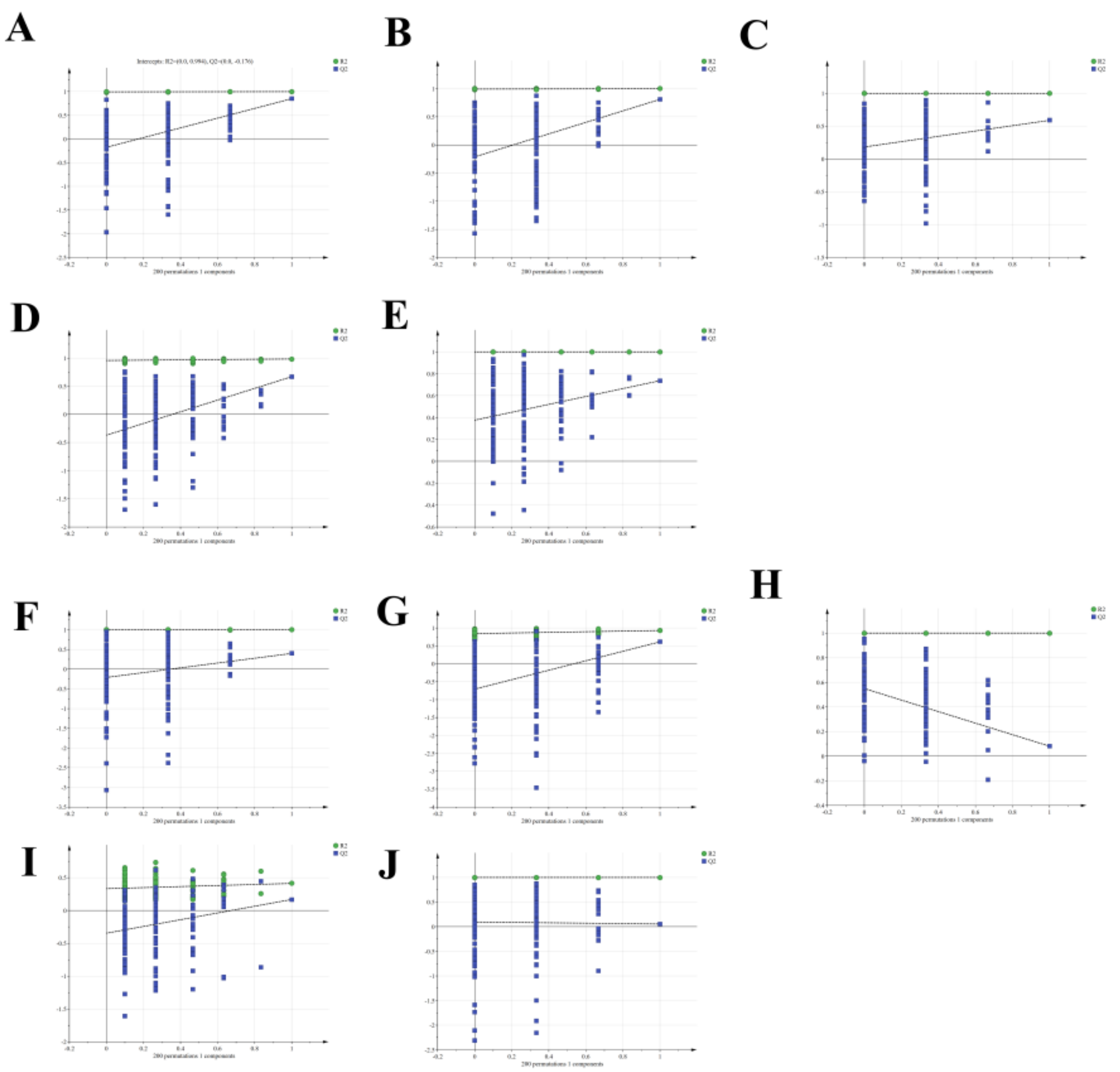

Fig.S1 Permutation test of each group and control group under positive and negative ions (A-E: positive ion mode, G-K: negative ion mode),control group vs. model group (A,G), H-SCSP group vs. model group (B,H), H-dSCSP group vs. model group (C,I), L-SCSP group vs. model group (D,J), L-dSCSP group vs. model group (E,K).

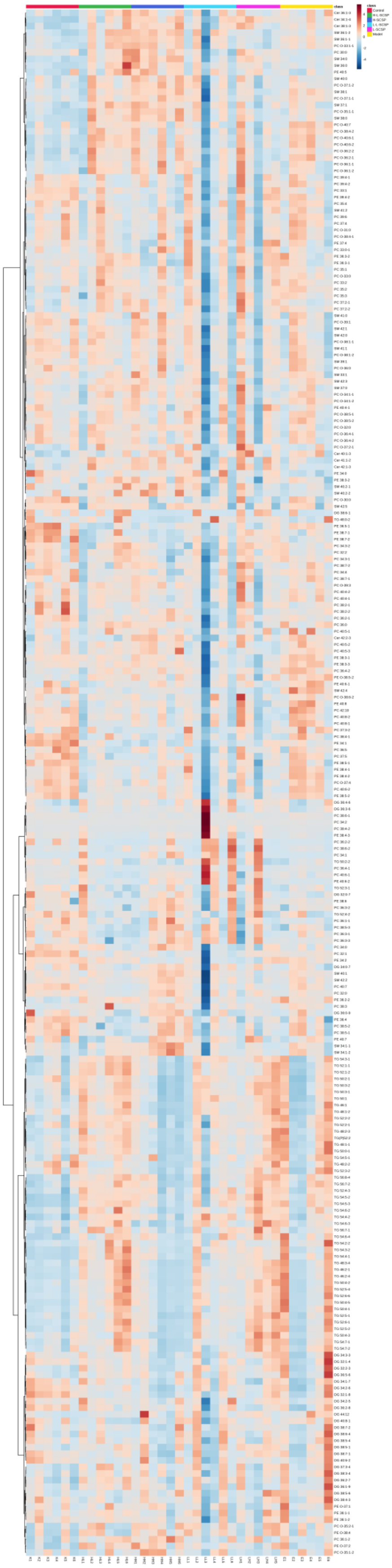

Fig.S2 Heat map of lipid metabolites difference between each group and model group(VIP>1).
